# From its roots come branches and growth for Disease Models & Mechanisms

**DOI:** 10.1242/dmm.048915

**Published:** 2021-01-24

**Authors:** Monica J. Justice

**Affiliations:** Program in Genetics and Genome Biology, The Hospital for Sick Children, and Department of Molecular Genetics, The University of Toronto, Toronto, Ontario M5G 0A4, Canada

In 2012, I received a call from Prof. Matthew Freeman, head of the William Dunn School of Pathology at the University of Oxford, asking if I was interested in a Senior Editor position at a relatively new The Company of Biologists’ journal called Disease Models & Mechanisms (DMM). Launched with Vivian Siegel as the first Editor-in-Chief (EiC), the vision was that the journal would grow to fill a niche for translational studies related to human disease that were carried out in model organisms. Ross Cagan, a fruit-fly researcher, and I were to become DMM's first research-active Senior Editors, starting in January 2013. It was to be an upward, but rewarding, climb; in spite of the success of the Company's other journals, including Development, Journal of Cell Science and Journal of Experimental Biology, nurturing a new journal into healthy growth is not easy.

**Fig. 1.**
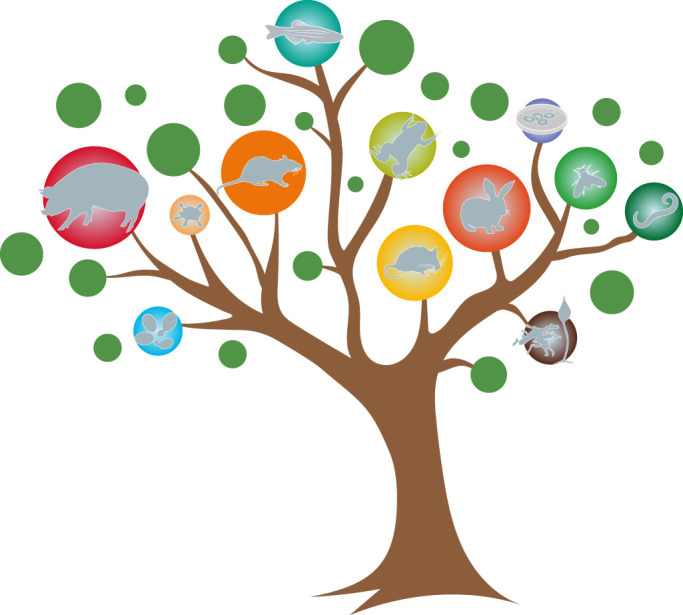
**Tree of model systems covered by DMM, including mouse, fish, worm, rat, fruit fly, rabbit, pig, slime mould, organoids, frog, yeast and human cell lines.**

DMM's roots lie in a broad range of scientific topics, and many different models (Fig. 1). Indeed, one of the strengths of DMM is its breadth of model organism coverage – studies using yeast, worm, slime mould, fly, fish, mouse, rat, rabbit, dog, pig and sheep as models have been published. At journal launch, Matthew and one of DMM's advisors, Daniel St Johnston, thought that the journal would receive primarily fruit-fly papers. However, submissions using mice for translational research have been the most prevalent. When I moved from Senior Editor to EiC in 2016, we added two academic Editors to help handle these mouse papers – Pamela Hoodless and Steven Clapcote, whom I thank for their dedication. During my tenure as EiC, we have seen increasing use of human induced pluripotent stem cells and organoids or 3D cultures as surrogates for human disease; thus, DMM has grown from model *organisms* to model *systems*.

To branch out into different scientific fields, DMM publishes special issues that cover one of a wide range of health-related topics, or focus on a certain model system, with expert Guest Editors determining the content. Our special issues have covered disease or health-related topics including neurodegeneration, neuromuscular disorders, drug discovery and cancer metabolism. We have also published special collections with a ‘spotlight’ on the rat, worm, fish and flies. DMM Scientific Editor Julija Hmeljak and I wrote an editorial on how model organisms have helped to diagnose and understand rare diseases, leading to a special collection on rare disease research (Hmeljak and Justice, 2019). Our next special issue will be on disorders of the RAS pathway, and strategies to treat them (find out more at https://dmm.biologists.org/content/ras).

I realised early on that rigorous standards for scientific reporting, and fast but realistic academic standards for manuscript handling, would eventually yield a vital solid root system for the journal. At a time when mouse models were under fire, Paraminder Dhillon, then DMM Reviews Editor, and I wrote a report on how to improve the validity and reproducibility of clinical translation (Justice and Dhillon, 2016). This was followed by a discussion on the importance of zebrafish research by DMM Editors Liz Patton and David Tobin (Patton and Tobin, 2019). Although some authors were naturally disappointed when their articles were rejected, our focus on standards has resulted in increasingly relevant and rigorous submissions. In 2018, we launched the ‘outstanding paper prize’. The selection is becoming increasingly competitive, and it is difficult to choose just one. Consistent with the scope of the journal, these outstanding papers cover a wide range of topics, including infectious disease in fish (Zhou et al., 2018) and cancer in flies (Bailetti et al., 2019). We look forward to announcing the 2020 winner in due course.

The Company of Biologists is a not-for-profit publishing establishment that is run by scientists, for scientists (see Box 1 for ways in which we support our community). What a unique concept in a world of big box publishing companies. Each journal funds topically appropriate Travelling Fellowships to enable researchers to visit other laboratories to learn a technique or to perform work not possible in their home laboratory. To raise awareness of DMM and further support our community, we launched Conference Travel Grants in 2015, allowing early-career researchers and independent Principal Investigators (PIs) without funding to attend meetings, conferences and courses relating to the areas of research covered by the journal. The success of these grants is a bit overwhelming: we have, to date, awarded 234 conference travel grants to applicants around the world, which, despite a doubling of the funding provided by The Company of Biologists, represents less than half of the applications we receive (Nicholson and Hmeljak, 2020).

**Box 1. Supporting DMM's communities.**

**Supporting our authors**

Over the years, DMM has undertaken numerous initiatives to improve the experience of authors, including partnering with Dryad to improve data accessibility, permitting format-free submission, adopting continuous publication (enabling speedier publication of the version of record), joining the Review Commons initiative and offering play-in-place movies.

**Supporting preprints**

DMM has long had preprint-friendly policies and, since 2016, has had a two-way portal between DMM and bioRxiv. Authors submitting to DMM can simultaneously deposit their article in bioRxiv, and vice versa.

**Supporting early-career researchers**

Outstanding paper prize

The DMM outstanding paper prize was set up to inspire young researchers embarking on their scientific careers. Each year, the journal awards a prize ($1000) to the first author of the paper that is judged by the journal's editors to be the most outstanding contribution to the journal that year.

DMM Conference Travel Grants

Launched in November 2015, DMM offers Conference Travel Grants of up to £600 to early-career researchers wanting to attend meetings and courses relating to the areas of research covered by the journal. We also welcome applications from independent group leaders and PIs with no independent funding.

DMM Travelling Fellowships

DMM also offers Travelling Fellowships of up to £2500 to graduate students and postdoctoral researchers wishing to make collaborative visits to other laboratories. These are designed to offset the cost of travel/accommodation and other related expenses.

First Person interviews

In our popular ‘First Person’ interviews, the first authors of Research articles published in DMM talk about their work in and out of the lab, the journeys that led them to where they are now and the issues they feel are priorities for early-career researchers.

Early-career Editorial Board members

In 2019, DMM appointed new members to its Editorial Board, with the aim of increasing its diversity in terms of career stage. We were delighted to announce the appointment of the following: Lukas Dow (Weill Cornell Medicine, New York, NY, USA), Susumu Hirabayashi (MRC London Institute of Medical Sciences, Imperial College London, London, UK), Yun Li (The Hospital for Sick Children, Toronto, Canada), Rachel K. Miller (McGovern Medical School, Houston, TX, USA) and Eirini Trompouki (Max Planck Institute of Immunobiology and Epigenetics, Freiburg, Germany).

**Supporting our reviewers**

Cross-referee commenting

DMM operates cross-referee commenting, whereby we invite referees to comment on the other referee reports before the editor makes a decision. The aim of this ‘cross-referee commenting’ step is to help resolve differences between referees, identify unnecessary or unreasonable requests, or – conversely – highlight valid concerns raised by one referee but overlooked by others.

Partnership with Publons

DMM's partnership with Publons allows reviewers to easily track and verify every review by choosing to add the review to their Publons profile when completing the review submission form.

**Supporting Open Access**

DMM has been awarded the Directory of Open Access Journals (DOAJ) seal. This is a “mark of certification for Open Access journals, awarded by DOAJ to journals that achieve a high level of openness, adhere to Best Practice and high publishing standards”. All DMM articles are published under the CC-BY license and deposited in PMC. DMM grants full article publication charge (APC) waivers for corresponding authors based in low-income and lower-middle-income economies and for other authors who genuinely have no funds to cover APCs. In 2019, all applications for an APC waiver fitted the criteria for approval and were granted a full or partial waiver.

In 2019, I had the privilege of hosting the first ever DMM Journal Meeting – with co-organisers Len Zon, Nancy Speck and Paresh Vyas – called ‘Blood Disorders: Models, Mechanisms and Therapies’. Blood disorders were chosen as our first topic for the meeting series because researchers in the field of haematopoiesis have made significant progress in advancing therapies developed from model organism research to the clinic. These advances, including bone marrow transfers, stem cell replacements and immunotherapies, have also had a large impact on the treatment of non-blood disorders. Held in the Joseph B. Martin Conference Center, embedded in the Harvard University campus, the meeting brought together diverse front-line researchers, stimulated discussions across basic research and clinical disciplines, and fostered collaborative links among researchers (Justice et al., 2020).

The Company of Biologists also hosts unique Workshops that bring together researchers that might not otherwise find each other to collaborate. As EiC, one of my benefits was to be able to attend (at least one) Company-sponsored workshop each year. One of my favourite workshops was ‘Understanding human birth defects in the genomic age’ (Khokha et al., 2020). The success of this Workshop led to the idea for a new Journal Meeting, which evolved to become ‘Developmental disorders: from mechanism to treatment’, jointly hosted with our sister journal Development. This meeting, organised by myself, James Briscoe (EiC of Development), Phil Beales and Lee Niswander, will hopefully be held outside Barcelona in fall 2021, COVID-19 permitting (https://www.biologists.com/meetings/mechanismtotreatment2021/). The meeting aims to bring together developmental biologists, human geneticists and clinical researchers who are united in the goal of understanding and treating developmental disorders.

Endeavouring to make my job as EiC of DMM more of a breeze than many might believe possible are the wonderful staff at The Company of Biologists’ Bidder Building in Cambridge, UK. Managing Editors Kirsty McCormack and, more recently, Rachel Hackett work calmly and steadily to ensure a smooth workflow. Claire Moulton, the publisher, is a force, with whom I share a particular vice that is not mentioned here. Reviews/Scientific Editors Sarah Allen, Paraminder Dhillon and Julija Hmeljak (in chronological order) have been an essential source of knowledge and support, in addition to their main role in crafting DMM's informative and timely ‘front section’. Each submission received the careful attention of administrators and, further downstream, copyeditors, with special mention to Debbie Thorpe, Laura Mason, Melissa Ray and Jodie Houghton, as well as the support of illustrators, marketing staff and the production team. The knowledge and experience I gained during my time as Senior Editor and EiC at DMM is invaluable: working within a not-for-profit community publishing environment is a joy.

Now, I am thrilled to pass the torch, after an exhaustive recruitment process led by the Board of Directors of The Company of Biologists, to Prof. Elizabeth (Liz) Patton (Vyas, 2020). Liz has been a Monitoring Editor with DMM since 2013, so her experience with the journal will branch into new energy and vision for the future (to be outlined in a future editorial). I leave DMM in very capable hands. Now, I will remain a Monitoring Editor, at least in the near future. Thank you to The Company of Biologists, DMM staff and my Editor colleagues for this wonderful experience!
